# THE IMPACT OF MEANINGFUL VOLUNTEER ENGAGEMENT IN AGING ADULTS: THE BALTIMORE EXPERIENCE CORPS TRIAL

**DOI:** 10.1093/geroni/igz038.2928

**Published:** 2019-11-08

**Authors:** Michelle C Carlson, George Rebok

**Affiliations:** 1 Johns Hopkins Bloomberg School of Public Health, Baltimore, Maryland, United States; 2 The Johns Hopkins University, Baltimore, Maryland, United States

## Abstract

Experience Corps was designed to embed cognitive, social, and physical activity into volunteer service by training older adults to serve in neighborhood elementary schools as mentors of children in Kindergarten-3 for 15 hours a week over two academic years. We incorporated cognitive activities through the intentional design of a variety of roles in reading, math, library support, and positive communication. Socially, volunteers engage with other volunteers, teachers, and children, and functional walking 3-4 days/week to and from as well as within the schools. The Baltimore Experience Corps Trial (BECT) is the largest randomized controlled trial (N = 702) examining the impact of volunteer engagement on cognitive functions in cognitively intact older adults, over sampling African Americans (91%) who have twice the risk of Alzheimer’s disease as whites. Findings will be summarized and demonstrate the dose-dependent cognitive, psychosocial, lifestyle activity, and brain benefits of volunteering for up to two years.

